# A comprehensive analysis and annotation of human normal urinary proteome

**DOI:** 10.1038/s41598-017-03226-6

**Published:** 2017-06-08

**Authors:** Mindi Zhao, Menglin Li, Yehong Yang, Zhengguang Guo, Ying Sun, Chen Shao, Mingxi Li, Wei Sun, Youhe Gao

**Affiliations:** 1 Department of Pathophysiology, Institute of Basic Medical Sciences, Chinese Academy of Medical Sciences/School of Basic Medicine, Peking Union Medical College, Beijing, China; 2 Department of Laboratory Medicine, Beijing Hospital, No.1 DaHua Road, Beijing, 100730 China; 3 State Key Laboratory of Bioactive Substance and Function of Natural Medicines, Institute of Materia Medica, Peking Union Medical College & Chinese Academy of Medical Sciences, Beijing, China; 4 Core Facility of Instrument, Institute of Basic Medical Sciences, Chinese Academy of Medical Sciences/School of Basic Medicine, Peking Union Medical College, Beijing, China; 5 Department of Nephrology, Peking Union Medical College Hospital, Peking Union Medical College and Chinese Academy of Medical Sciences, Beijing, China; 6 Department of Biochemistry and Molecular Biology, Beijing Normal University, Gene Engineering and Biotechnology Beijing Key Laboratory, Beijing, China

## Abstract

Biomarkers are measurable changes associated with the disease. Urine can reflect the changes of the body while blood is under control of the homeostatic mechanisms; thus, urine is considered an important source for early and sensitive disease biomarker discovery. A comprehensive profile of the urinary proteome will provide a basic understanding of urinary proteins. In this paper, we present an in-depth analysis of the urinary proteome based on different separation strategies, including direct one dimensional liquid chromatography–tandem mass spectrometry (LC/MS/MS), two dimensional LC/MS/MS, and gel-eluted liquid fraction entrapment electrophoresis/liquid-phase isoelectric focusing followed by two dimensional LC/MS/MS. A total of 6085 proteins were identified in healthy urine, of which 2001 were not reported in previous studies and the concentrations of 2571 proteins were estimated (spanning a magnitude of 10^6^) with an intensity-based absolute quantification algorithm. The urinary proteins were annotated by their tissue distribution. Detailed information can be accessed at the “Human Urine Proteome Database” (www.urimarker.com/urine).

## Introduction

Urine is associated with glomerular filtration, tubular reabsorption and secretion^1^. Biomarkers are measurable changes associated with the disease^2^. Because urine can accumulate changes from the body^2, 3^, it is considered to be one of the most attractive sources for early and sensitive biomarker discovery. Urinary proteomic studies have identified many candidate biomarkers for various urogenital diseases, such as acute kidney injury, bladder cancer and diabetic nephropathy^4–6^. As urinary proteins are composed largely of filtered plasma proteins, the urinary proteome is also considered to be valuable for detecting a broad range of complex disorders, such as encephalopathy, heart failure and intestinal ischemia^7–9^.

In the biomarker discovery process, it is essential to comprehensively profile the normal urinary proteome as a baseline reference. With the rapid development of mass spectrometry (MS), larger numbers of urinary proteins were identified by various strategies. In 2001, Patterson *et al*. first identified 124 urinary proteins^10, 11^. In 2005, Sun *et al*. identified 226 proteins in normal urine with quality control LC/MS/MS data^3^. In 2006, Adachi *et al*. reported the first urinary proteome result (1543 proteins) from high resolution mass spectrometry^12^. In 2011, 1823 urinary proteins were identified by high resolution MS and MS/MS^13^. Many efforts have been made to identify more urinary proteins in recent years^14–16^. Currently, the human urine PeptideAtlas database contains a total of 23,739 peptides corresponding to 2487 proteins^17^.

**Figure 1:**
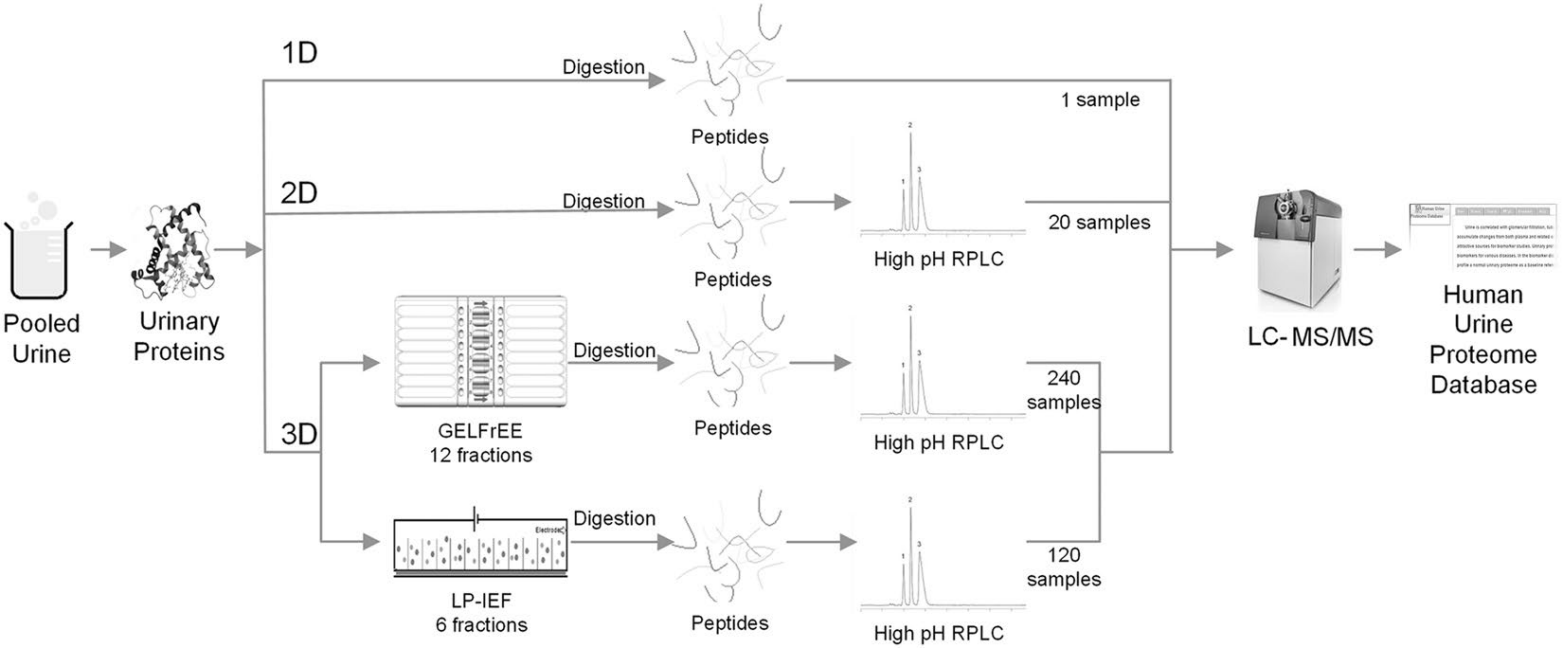
The workflow of urinary proteome analysis. Pooled urine from 24 humans was analyzed using three separation strategies. 1D: Urinary peptides were directly analyzed via 1DLC/MS/MS without fractionation. 2D: Urinary peptides were analyzed via offline RPLC and 1DLC/MS/MS. 3D: Urinary proteins were first fractionated by GELFrEE/LP-IEF prior to offline RPLC. A total of 383 fractions were analyzed by LC/MS/MS using high-resolution TripleTOF 5600 MS. A urine proteome database was then constructed based on bioinformatics analyses.

In 2014, two large-scale MS-based drafts of the human proteome identified 17,294 and 18,097 human gene products from 30 and 44 tissues and body fluids, respectively^18, 19^. In each study, the number of identified proteins was quite large and even approached the number of protein-coding genes in the complete human genome analysis^20^. Compared with the depth of the human tissue proteome, the urinary proteome has been relatively less studied. We are curious about how many proteins could be identified in human urinary proteome. Therefore, we performed an in-depth urinary proteomic analysis using normal human urine samples. And to achieve maximal urinary proteome coverage one-, two- and three-dimensional separation strategies (Fig. 1) were employed in this study. By in-depth analysis, a readily obtainable source for the human urinary proteome, “Human Urinary Proteome Database” could be provided. The comparison of three separation strategies could provide detailed information about the potential application of different separation methods. The detailed workflow was as followings: In one-dimensional (1D) separation, digested urinary peptides were directly analyzed by 1D liquid chromatography-tandem mass spectrometry (LC/MS/MS). In two-dimensional (2D) separation, urinary peptides were fractionated by offline high-pH reverse-phase liquid chromatography (RPLC) prior to analysis by 1DLC/MS/MS. In three-dimensional (3D) separation, urinary proteins were first fractionated by gel-eluted liquid fraction entrapment electrophoresis (GELFrEE) or liquid-phase isoelectric focusing (LP-IEF) and urinary peptides digested from GELFrEE/LP-IEF fractions were fractionated by RPLC as performed for 2D separation and finally analyzed by 1DLC/MS/MS. In total, 383 LC/MS/MS runs were analyzed by hybrid quadrupole-time-of-flight mass spectrometry (TripleTOF 5600).

## Results and Discussion

### Comprehensive identification of urinary proteome

In this study, pooled urine samples were used to establish a large database of urinary proteins. The following filters were used to select the final protein identification list (1). The FDR at the protein level was set to <1%, and (2) each protein should include at least two unique peptides. When identified peptides were shared between two proteins, they were combined and reported as one protein group. The results from 1DLC, 2DLC and 3DLC yielded average FDRs of 0.10%, 0.26% and 1% at the spectrum, peptide and protein levels, respectively (Supplemental File 1). Then the datasets were combined together with Scaffold perSPECtives.

**Figure 2:**
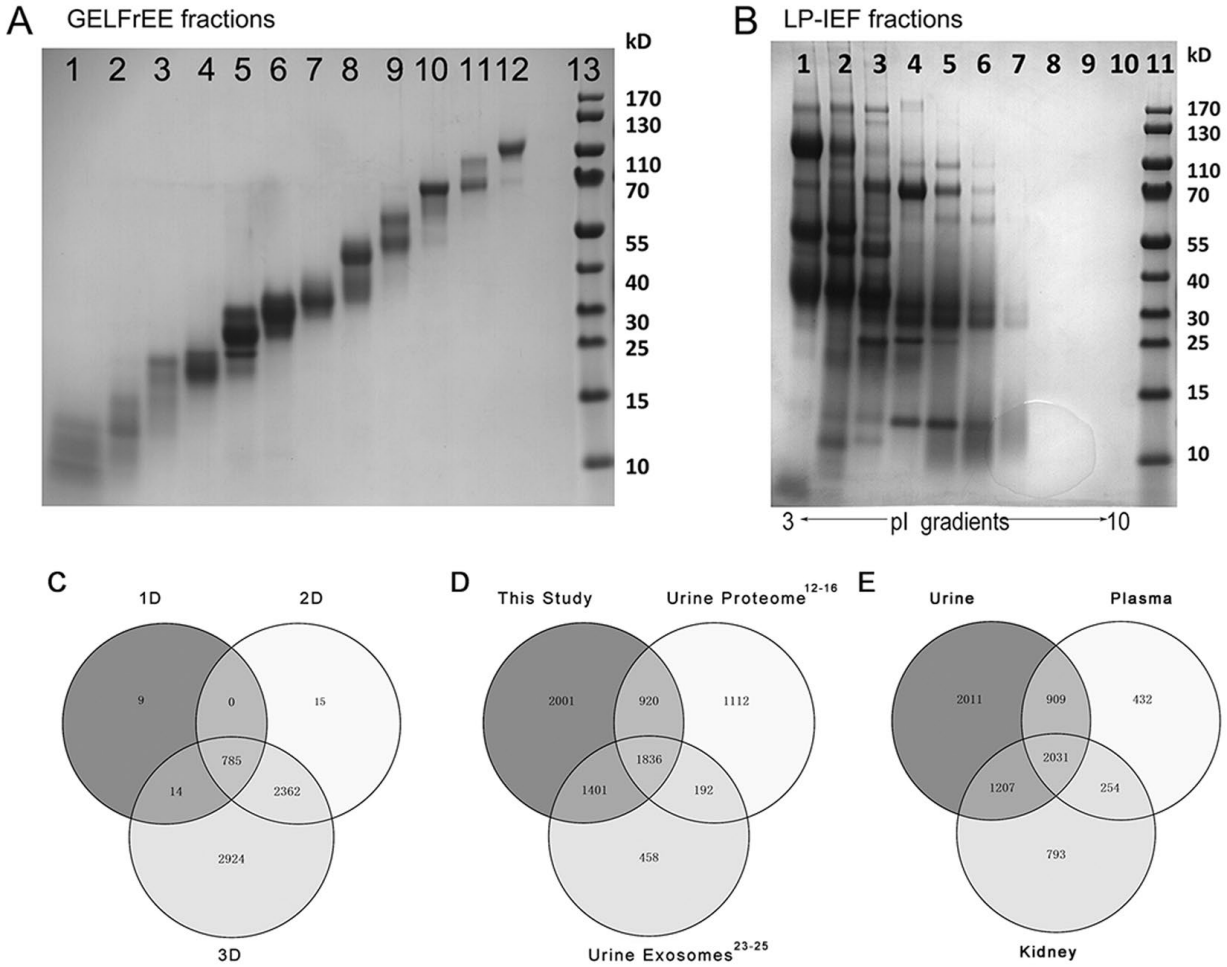
The results from three separation strategies. (**A**) A Coomassie-stained Bis-Tris gel image of 12 GELFrEE fractions over a broad mass range. (**B**) Coomassie-stained Bis-Tris gel image of 10 LP-IEF fractions over a pI range from 3 to 10. (**C**) Venn diagram of proteins identified by three separation strategies. (**D**) Venn diagram of proteins identified from this study as well as previous urine and exosome proteome studies. (**E**) Comparative analysis of the urine, kidney and plasma proteome.

In 1D analysis, 808 protein groups were identified in three technical replicates, and the protein-overlapping rate was 86.3%, indicating the superior reproducibility of LC/MS analysis. In 2D analysis, a total of 3162 protein groups were identified. In 3D analysis, urinary proteins were first separated by GELFrEE/LP-IEF (Fig. 2A,B). GELFrEE enables mass range proteome separations based on molecular weight (MW), and IEF fractionates proteins according to isoelectric point (pI)^21, 22^. The GELFrEE and LP-IEF fractions were then further separated by RPLC, and a total of 6085 protein groups were identified. The overlap among the proteins identified in the 1D, 2D and 3D analyses is displayed in Fig. 2C. Almost all proteins from the 1D and 2D analyses were included in the 3D results except for 9 and 15 proteins from the 1D and 2D results, respectively. The possible reasons why these proteins cannot be identified in the 3D methods were still unknown. Maybe these proteins were lost during 2D or 3D separate by high pH RPLC or IEF/GELFrEE. It may be also as a result of the random sampling of DDA detection modes. Therefore, we are not sure whether these proteins were false positive identification or not. Then these proteins (Supplemental File 2) were removed from the subsequent analysis to ensure data accuracy and reliability. Thus, the whole urine proteome dataset eventually contained 6085 protein groups (Supplemental Table 1).

Several studies have been conducted to characterize the normal human urinary proteome. Table 1 summarizes the current largest-scale studies of human urine and urinary exosomes using high-resolution MS^12–16, 23–25^. The protein accessions in each dataset were mapped to the corresponding gene IDs^26, 27^. Total nine large-scale urinary and exosome proteomic analyses were performed in recent years. When all of the data from these nine studies were combined, a total of 8021 gene products were detected in the human urinary proteome (Supplemental Table 2). When comparing previous data with our results (Fig. 2D), total 2001 gene products were uniquely identified in this study. The possible reasons of differences in urine proteome between different studies may be genetic factors, individual variations, different separate methods and MS preference.

**Table 1:** Recent large-scale proteomic studies of healthy human urine.

Sample	Number of identifications	Database	MS Instrument	Analysis Methods	Single peptide included	Reference
Urine	1543	IPI	LTQ-Orbitrap	SDS-PAGE	Yes	Adachi *et al*.^12^
Urine	1310	IPI	LTQ-Orbitrap	SCX/SAX	Yes	Li *et al*.^14^
Urine	1823	GI	LTQ-Orbitrap Velos	SDS-PAGE	Yes	Marimuthu *et al*.^13^
Urine	1985	IPI	LTQ-Orbitrap Velos	SDS-PAGE	Yes	Zheng *et al*.^15^
Urine	3429	Uniprot	LTQ-Orbitrap Velos Pro	Combinatorial peptide ligand libraries	Yes	Santucci *et al*.^16^
Exosome	1132	GI	LTQ	SDS-PAGE	Yes	Gonzales *et al*.^23^
Exosome	3280	Uniprot	LTQ-Orbitrap Velos	SDS-PAGE followed by SCX	No	Wang *et al*.^24^
Exosome	1830	Swissprot	LTQ-Orbitrap Velos	SDS-PAGE	No	Hogan *et al*.^25^
Urine	6085	Swissprot	TripleTOF 5600	GELFREE/IEF-RPLC	No	Zhao *et al*. 2017

Urinary proteins, which are considered to represent the protein composition of the output of the kidneys^28^, are primarily composed of proteins derived from plasma filtration and urinary tract system secretion. A comparative analysis of the urine, plasma and kidney proteome would provide a more concrete link to determine how many plasma- and kidney-related proteins could be detected in urine. The PeptideAtlas builds yielded 3553 and 4005 non-redundant proteins at 1% FDR for the plasma and kidney proteomes^29^. In contrast, a total of 2940 (47.7%) and 3238 (52.6%) of the gene products identified in this urinary proteome study were common to the gene products (Fig. 2E) that were reported in the plasma (81.1%) and kidney proteomes (75.6%), respectively. According to previous report, approximately 30% of urinary proteins originate from the plasma proteins, whereas 70% comes from the kidney and the urinary tract^30^. From our study, maybe the difference between plasma and urine is smaller than expected. And it might be possible that more common proteins will be identified with the development of MS in the future. By comparison with kidney proteome, we want to show the overlap between urine and kidney proteome. The large overlap may give evidences that urine can better reflect the functions of kidney.

### Quantitative analysis of urinary proteins

**Figure 3:**
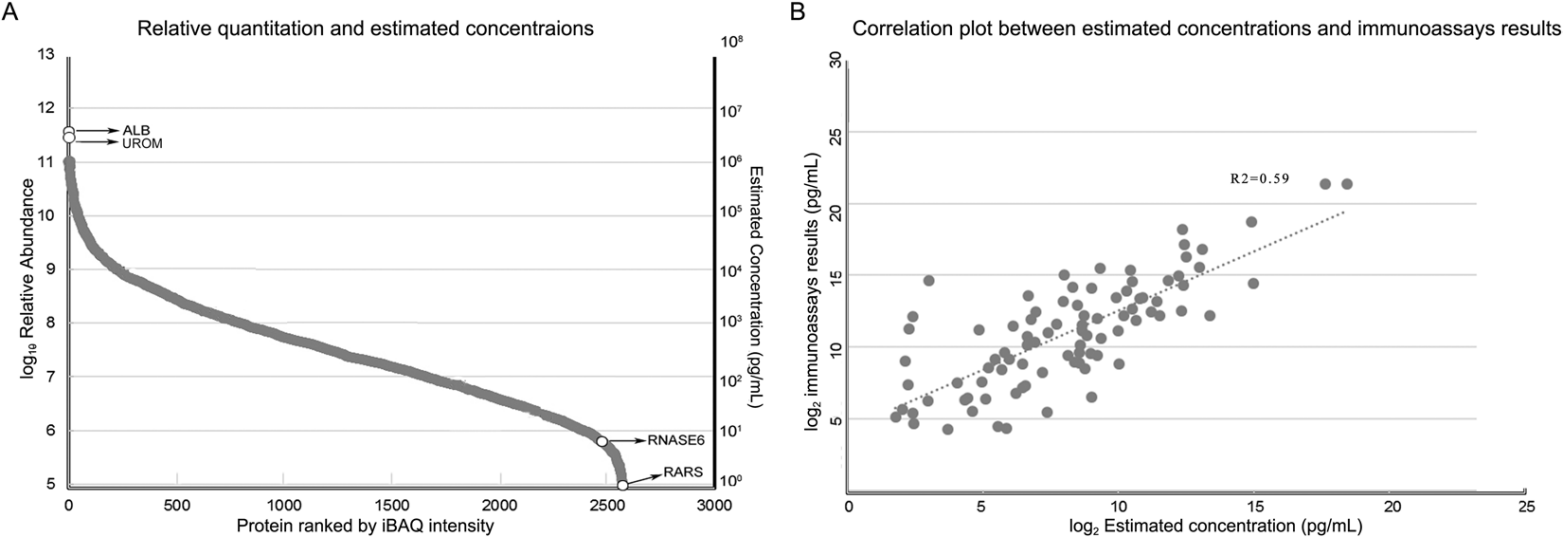
Quantitative analysis of urinary proteins by the iBAQ method. (**A**) The relative expression and concentrations of 2,571 proteins in the 2D analysis were estimated by iBAQ. The left y-axis represents relative abundance, and the right y axis represents estimated concentration (pg/mL). (1) ALB: albumin; UROM: uromodulin, the two most abundant proteins. (2) RARS: arginine-tRNA ligase, the least abundant protein in 2D analysis; (3) RNASE 6: ribonuclease K6, the least abundant protein in 1D analysis. (**B**) Correlation plot between estimated concentrations and immunoassays results.

Quantitation of urinary proteins will improve our understanding of the urinary proteome and will facilitate the development of urinary biomarkers. Accordingly, we aimed to quantify each protein using the iBAQ algorithm, which provides a rough indication of actual protein levels^31^. In 3D analysis, equal amounts of protein from each GELFrEE/LP-IEF fraction were used for LC/MS/MS analysis, which thus cannot provide an accurate quantitative analysis. Therefore, data from the 2D strategy were used, and a total of 2571 proteins were quantified with the iBAQ algorithm. The dynamic range of relative abundance spanned six orders of magnitude (Fig. 3A), which was consistent with previous observations^32^. Considering that more than 3000 proteins in the 3D analysis were not quantified, the dynamic range is expected to be even bigger. In the present analysis, serum albumin and uromodulin were the most abundant urinary proteins.

The average concentration of urinary albumin, which was one of the most easily detected urinary proteins, was approximately 2.2–3.3 µg/mL^12, 33^ in normal human urine. With the iBAQ value ratios, concentrations of the other 2570 proteins could subsequently be estimated (Supplemental Table 3). The estimated concentration of arginine-tRNA ligase (RARS), which had the lowest relative abundance in the 2D analysis, was 0.68~1.02 pg/mL. As the 2D results contained almost all of the proteins from the 1D separation, concentrations of the 753 proteins from the 1D analysis could be inferred. Among them, ribonuclease K6 (RNASE 6) was the least abundant protein in the 1D analysis with an estimated concentration of 5.58~8.37 pg/mL.

To evaluate the accuracy of estimated concentrations with the iBAQ algorithm and the corresponding application to other samples in different labs, the estimated concentrations were compared with the results from immunoassay screening in a previous urinary candidate biomarker study^34^. A total of 89 proteins were commonly evaluated in both studies (Fig. 3B and Supplemental Table 4, R^2^ = 0.59).

### Functional annotation of three separation strategies

Functional annotations of urinary proteins based on the degree of analysis depth may be helpful in providing insight into the analysis approach difference in protein composition, reflecting pathophysiological states and determining suitable separation methods for some diseases. To analyze the protein identification data from the three separation strategies, 6085 proteins were divided into three groups as follows: Group 1D, proteins identified in 1D analysis (799 proteins); Group 2D, proteins identified in 2D analysis, excluding those identified in the 1D analysis (2362 proteins); and Group 3D, proteins identified in 3D analysis, excluding those identified in both the 1D and 2D analyses (2924 proteins).

**Figure 4:**
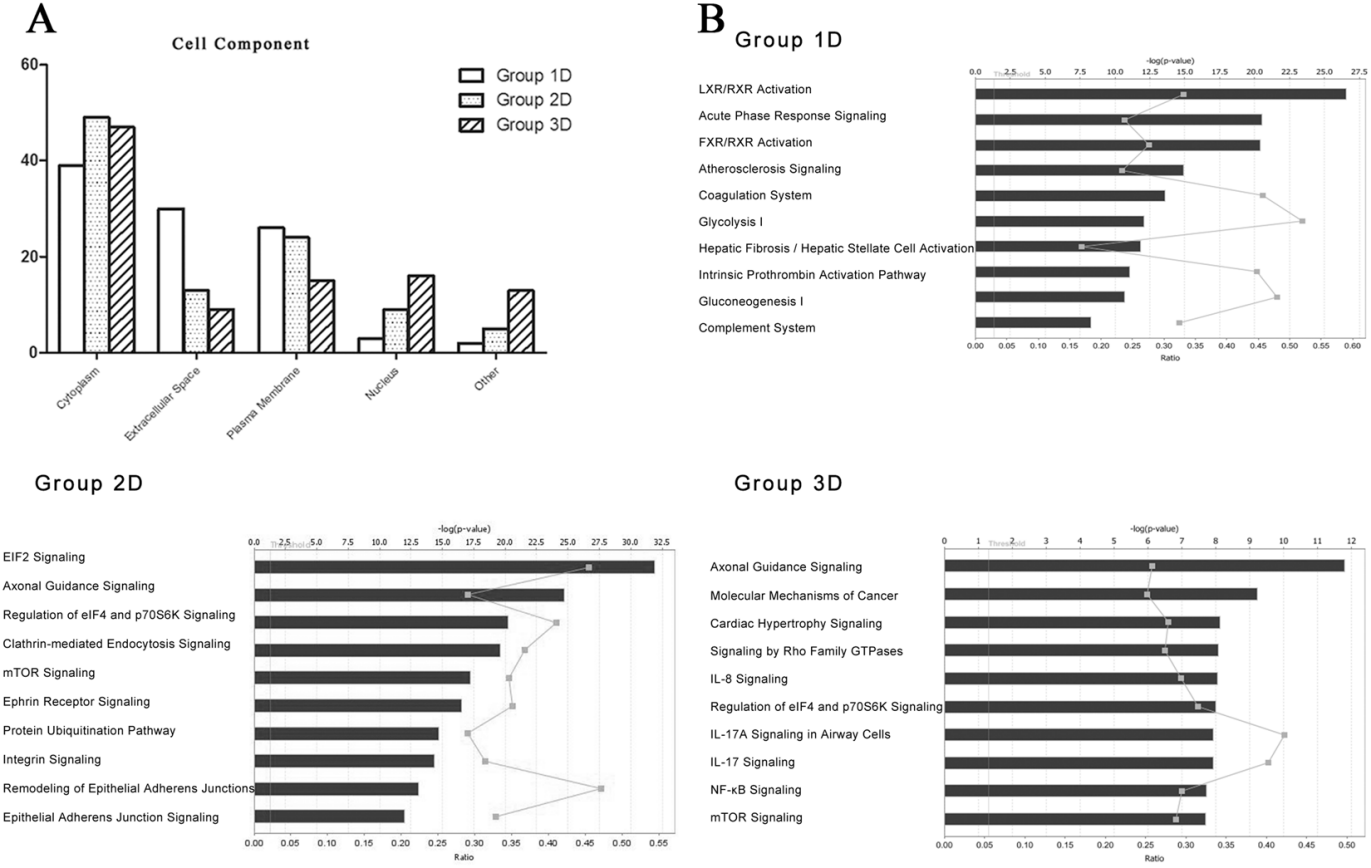
Cellular component and canonical pathway analyses of three separation groups. (**A**) Cellular component analysis of the three groups. (**B**) The top 10 canonical pathways from the three groups. The y-axis denotes the negative log of the p value.

IPA analysis was performed to provide insight into the functions of the three groups (Fig. 4 and Supplemental Table 5). Extracellular proteins and plasma membrane proteins were enriched in Group 1D (56%), as previously reported^12^. The most significant pathways in Group 1D were functionally similar to plasma components, such as inflammatory responses, coagulation and glucose metabolism. Acute phase response signaling, which is one of the top pathways for Group 1D, is a rapid inflammatory response that provides protection against some infections by nonspecific defenses. It consists of an increase in inflammatory factors (such as IL-1) and a change in the levels of several plasma proteins (such as ALB and APOA1/2). For example, alpha-1-acid glycoprotein 1 (ORM1), an extracellular protein, is involved in the acute phase response. Overexpression of ORM1 in urine was associated with acute pediatric appendicitis^35^. As Group 2D demonstrated considerable enrichment of intracellular proteins (58%), most of the pathways were involved in cellular signaling such as EIF2 Signaling and Regulation of eIF4 and p70S6K signaling. Proteins in Group 3D were also over-represented in the cytoplasm and nucleus (63%). Most of the canonical pathways in Group 3D were closely related to interleukin signaling.

Considering the above results, we assumed that proteins in the three groups were functionally different. If the purpose of research was to study basic physiological activities, such as cell movement and proliferation, maybe the urinary proteome can be analyzed without further separation in most cases. If aim at intracellular activities and functions of organs, maybe the in-depth analysis is necessary.

### Landscape of proteins detected in urine

Previous studies reported that urine might reflect kidney function and identified some potential biomarkers of kidney disease^36^. According to previous analyses, the Human Urinary Proteome Database contains proteins localized in the glomeruli of nephron segments (Table 2) and molecules to detect injures to specific tubules of nephron segments (Table 3). Extracellular macromolecular laminin, type IV collagen α3α4α5, heparan sulfate proteoglycan agrin, and nidogen were the main components of the glomerular basement membrane (GBM)^37^ and could all be identified by the one-dimensional method without fractionation. Nephrin and podocin are both specifically expressed in the slit diaphragm, which is pivotal in maintaining the selective permeability of podocytes in the glomerular filtration barrier^38^. The cytoplasmic protein CD2-associated protein (CD2AP) localizes to the podocyte slit diaphragm where it has been shown to bind to nephrin and podocin^39^. The above three podocyte-related proteins could be identified in Group 2D. The fatty acid-binding proteins (FABPs) in Group 2D are a class of small intracellular proteins that bind long chain fatty acids. Liver-type FABP is mainly present in the cells of the proximal tubules, while heart-type FABP is predominantly localized in the distal tubules^40^. The above results showed that both glomerulus and tubules-related proteins could be found in the urine, which indicated that the urine proteome might reflect changes of kidney function.

**Table 2:** Urinary candidate biomarkers of glomerular injury.

Protein Name	Uniprot ID	Protein in Group	Nephron segment^25, 37, 38, 55, 56^	Location	Molecular Function	Biomarker Application	Reference
Podocin	Q9NP85	2D	Podocyte & slit diaphragm	Plasma Membrane	other	IgA nephropathy, membranous nephropathy	57, 58
Alpha-actinin-4	O43707	1D	Podocyte	Cytoplasm	other	Diabetic nephropathy, focal segmental glomerulosclerosis	59, 60
Neprilysin	P08473	1D	Podocyte	Plasma Membrane	peptidase	Glomerulonephritis	61
Myosin-9	P35579	1D	Podocyte & mesangial cells	Cytoplasm	enzyme	Glomerulopathy	62
Agrin	O00468	1D	Glomerular basement membrane	Plasma Membrane	other	Diabetic nephropathy, transplant glomerulopathy	63, 64
Collagen alpha-3(VI) chain	P12111	1D	Glomerular basement membrane	Extracellular Space	other	Alport syndrome, diabetic nephropathy	65, 66
Nidogen	P14543, Q14112	1D	Glomerular basement membrane	Extracellular Space	other	Membranous nephropathy	67
Laminin	Multiple Ma	1D	Glomerular basement membrane	Extracellular Space	other	Diabetic nephropathy	68
Nephrin	O60500	2D	Podocyte	Plasma Membrane	other	Diabetic nephropathy	69
CD2-associated protein	Q9Y5K6	2D	Podocyte	Cytoplasm	other	Focal segmental glomerulosclerosis	70
Podocalyxin	O00592	1D	Podocyte & parietal epithelial cells	Plasma Membrane	kinase	Diabetic nephropathy	71
Vascular endothelial growth factor	P15692, P49767, P49765	3D	Podocyte	Extracellular Space	growth factor	Diabetic nephropathy	72
Proliferating cell nuclear antigen	P12004	3D	Parietal epithelial cells & podocyte	Nucleus	enzyme	Schistosomal nephropathy	73
Secretory phospholipase A2 receptor	Q13018	2D	Glomerulus	Plasma Membrane	transmembrane receptor	Idiopathic membranous nephropathy	41
Complement C3	P01024	1D	Glomerular basement membrane, mesangium, capillary loops	Extracellular Space	peptidase	Lupus nephritis	74
Apolipoprotein E	P02649	1D	Mesangial cells	Extracellular Space	transporter	Diabetic nephropathy	75
CD151 antigen	P48509	2D	Podocyte, glomerular basement membrane	Plasma Membrane	other	Type 1 diabetic nephropathy	76
Cofilin-1	P23528	1D	Podocyte	Nucleus	other	Hypertension-induced renal damage	77
Fibronectin	P02751	1D	Mesangial and subendothelial cells	Extracellular Space	enzyme	Glomerulopathy with fibronectin deposits	78
Myeloperoxidase	P05164	1D	Glomerular capillary	Cytoplasm	enzyme	Anti-neutrophil cytoplasmic antibody-associated glomerulonephritis	79

**Table 3:** Urinary candidate biomarkers of tubular injury.

Protein Name	Uniprot ID	Protein Group	Nephron segment^25, 40, 80^	Location	Molecular Function	Biomarker Application	Ref.
Beta-2-microglobulin	P61769	1D	Proximal tubule	Plasma Membrane	transmembrane receptor	Acute renal allograft rejection, acute kidney injury, diabetic nephropathy	81, 82
GST-alpha	P09210	1D	Proximal tubule	Cytoplasm	enzyme	Acute kidney injury	83
GSTP1	P09211	1D	Distal tubule	Cytoplasm	enzyme	Acute renal failure	81
Clusterin	P10909	1D	Proximal tubule & distal tubule	Cytoplasm	other	Renal-cell carcinoma, acute kidney injury	84
Cubilin	O60494	1D	Proximal tubule	Plasma Membrane	transmembrane receptor	Type 1 diabetes	85
Liver-type fatty acid-binding protein acid-binding protein	P07148	2D	Proximal tubule	Cytoplasm	transporter	Diabetic nephropathy, contrast nephropathy, IgA nephropathy	40
Heart-type fatty acid-binding protein	P05413	2D	Distal tubule	Cytoplasm	transporter	Acute kidney injury after cardiac surgery	86
Cystatin-C	P01034	1D	Glomerulus & proximal tubule	Extracellular Space	other	Acute kidney injury, acute renal dysfunction	87, 88
Calbindin	P05937	1D	Distal tubule & collecting duct	Cytoplasm	other	Distal nephron segment injuries	89
CYR61	O00622	2D	Proximal tubule	Extracellular Space	other	Glomerular disease	90
Alkaline phosphatase, tissue-nonspecific isozyme	P09923	2D	Proximal tubule	Plasma Membrane	phosphatase	Acute renal failure	91
Intestinal-type alkaline phosphatase	P05186	2D	Proximal tubule	Plasma Membrane	phosphatase	Diabetic nephropathy, acute renal failure	92
Alpha-N-acetylglucosaminidase	P54802	1D	Proximal tubule	Cytoplasm	enzyme	Acute kidney injury	93
Netrin-1	O95631	3D	Proximal tubule	Extracellular Space	growth factor	Acute kidney injury, diabetic nephropathy	94
Neutrophil gelatinase-associated lipocalin	P80188	1D	Proximal tubule & distal tubule	Extracellular Space	transporter	Acute kidney injury, chronic kidney disease	95
Osteopontin	P10451	1D	Proximal tubule & loop of henle & distal tubule	Extracellular Space	cytokine	Progressive renal injury	96
Interleukin-18	Q14116	2D	Proximal tubule	Extracellular Space	cytokine	Acute kidney injury	97
Retinol-binding protein	P02753, P82980, P50120, P09455	1D	Proximal tubule	Extracellular Space, Cytoplasm	transporter	Acute kidney injury, renal failure	98

Some tissue or serum biomarkers of kidney diseases could also be detected in our urine proteome database. For example, the phospholipase A2 receptor (PLA_2_R), a plasma membrane glycoprotein located on normal podocytes, was a major target antigen in idiopathic membranous nephropathy^41^. PLA_2_R could be detected in Groups 2D and 3D. Urokinase plasminogen activator surface receptor (uPAR) is a glycosylphosphatidylinoisitol -anchored three-domain protein and is expressed in human glomerular cells. Serum concentrations of soluble uPAR are significantly elevated in most subjects with primary focal segmental glomerulosclerosis (FSGS)^42^. If these tissue or serum biomarkers could be confirmed as urinary biomarkers, the human urinary proteome database would provide a convenient way to discover noninvasive urinary candidate biomarkers. In addition to kidney diseases, previous studies also reported that some other diseases, such as acute pancreatitis^43^, might possess urinary biomarkers. The human urinary proteome database provides brief information on known biomarkers for predicting various types of organ injury (Supplemental Table 6).

**Figure 5:**
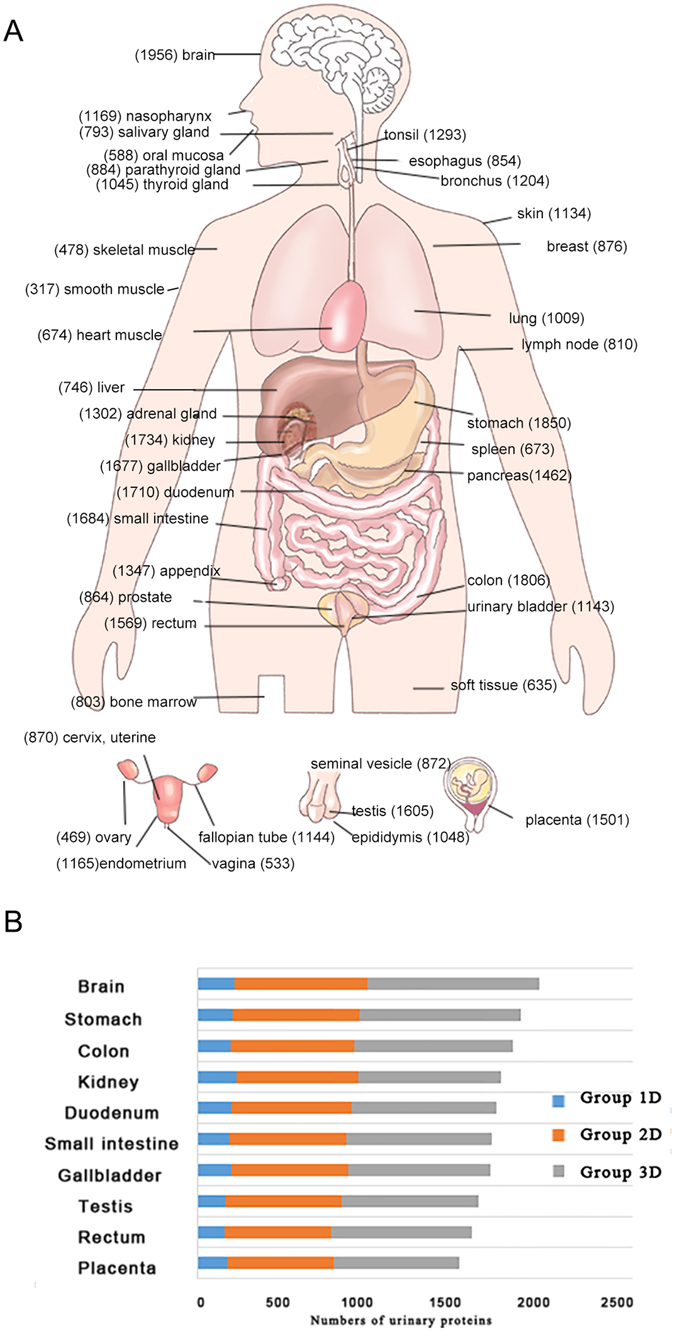
Tissue distribution of urinary proteins at protein level. (**A**) Urinary proteome distributions across 44 tissues. The numbers in the bracket denote the number of highly expressed proteins of the tissue detected in urine. (**B**) The distribution of tissue-related proteins and the corresponding separation strategy for top ten tissues.

Moreover, these proteins detected in urine were annotated by their tissue distribution based on an integrated omics approach that involves quantitative transcriptomics and tissue microarray–based immunohistochemistry in previous studies^44^. The detailed annotation data of each protein were shown in the following database. The tissue with maximum numbers of highly expressed proteins detected in urine both at protein and mRNA levels was brain (Fig. 5A, Supplemental Figure 1). Other tissues with more highly expressed proteins were mostly digestive organs such as colon and stomach. As expected, more tissue-related proteins could be detected in Group 2D and 3D than in Group 1D (Fig. 5B).

### The Human Urinary Proteome Database

**Figure 6:**
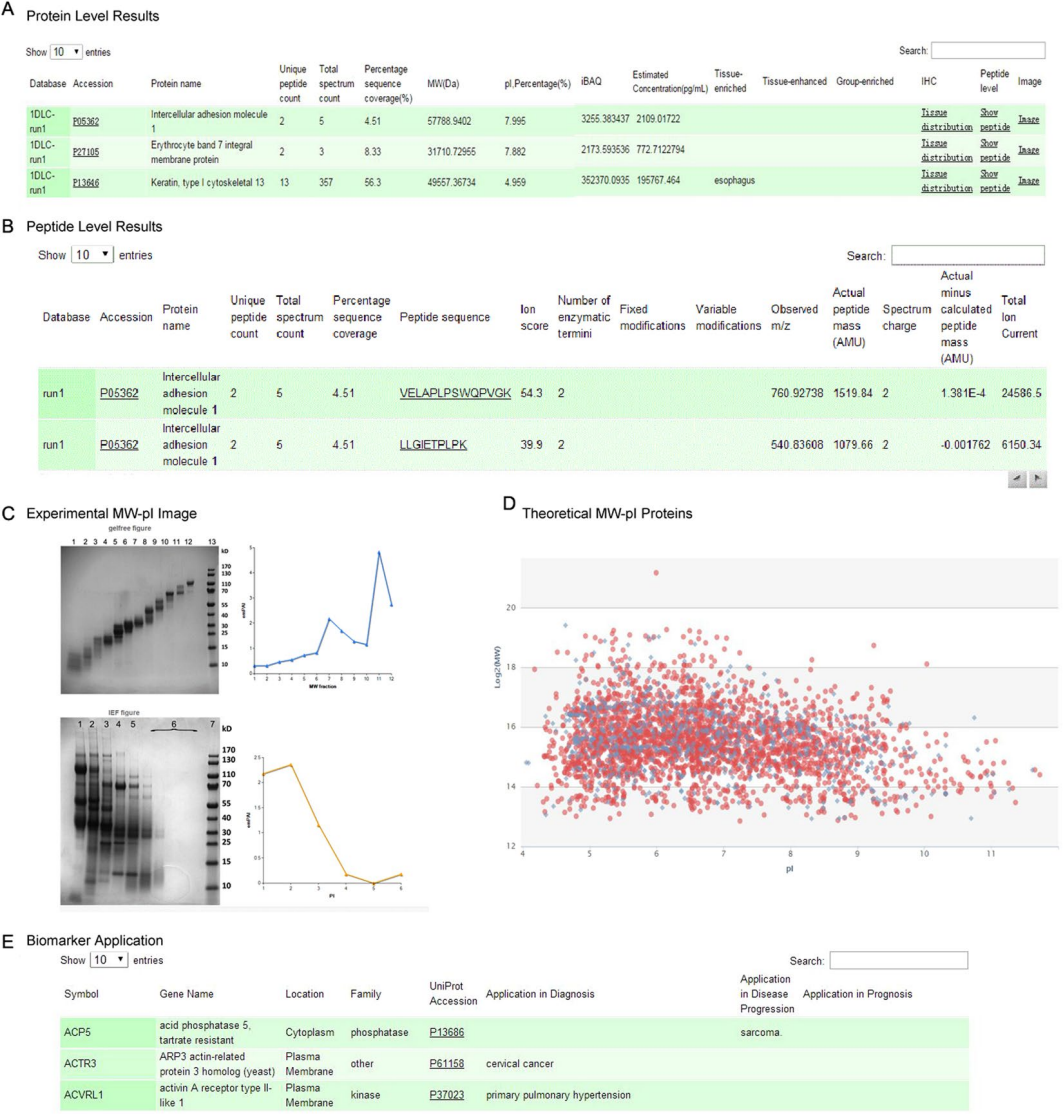
An overview of the human urinary proteome database. (**A**) The protein level results include the unique peptide count, total peptide count and relative quantitation and estimated concentration. Proteins are linked to the UniProt website by clicking the accessions. (**B**) The peptide level results include peptide sequences and observed m/z values. (**C**) The database provides the experimental pI and MW distribution of all identified proteins. (**D**) The “MW-PI” section provides a succinct figure summarizing the theoretical MW and pI information for each protein. (**E**) Biomarker application of all identified proteins.

To provide a readily obtainable source for the human urinary proteome, the “Human Urinary Proteome Database” was constructed (Fig. 6) based on the above analyses. The database was constructed using open source technologies and is freely available at www.urimarker.com/urine. A total of 3048648 spectra, 68151 unique peptides and 6085 proteins are included, along with detailed information such as the protein name, accession number, peptide sequence, sequence coverage and unique peptide number.

Each protein is featured with annotated data, including relative quantitative information, estimated concentrations, theoretical and experimental MW and pI. Remarkably, some high-abundance proteins were observed spanning multiple fractions in both the GELFrEE and LP-IEF separations. It is generally accepted that mass/pI deviation may occur due to the presence of fragments, protein polymers, isoforms, protein degradation, post-translational modifications and low focusing quality in the basic region of the immobilized pH-gradient strips, as well as due to the pI prediction algorithm used^45–48^. Moreover, a novel section labeled ‘MW-PI image’ provides a succinct figure indicating the significant MW and pI information for all of the identified urinary proteins, which might be helpful for generating a brief scan of proteins in a pI and MW range of interest. For biomarker studies, the “Biomarker” section also yields potential biomarkers for applications in diagnosis, disease progression and prognosis.

The Human Urinary Proteome Database serves as a reference repository for urinary proteins, as it offers the largest number of such proteins to date. All of the data retrieved from three separations not only detail the normal human urinary proteome but also categorize all proteins by different separation methods. Moreover, the database can be used for targeted proteomics that rely on the proper selection of peptides and transitions to guide the selection of proteotypic peptides for candidate proteins^49^.

## Materials and Methods

### Ethics statement

Prior to study enrollment, all of the healthy volunteers were given a verbal explanation of the study and each participant signed an informed consent document. The consent procedure and the research protocol were approved by the Medical Ethics Committee of Peking Union Medical College. All methods in this study were performed in accordance with the guidelines and regulations.

### Experimental design and statistical rationale

Twenty-four healthy volunteers (38 ± 11 years old), including twelve males and twelve females, were enrolled. Exclusion criteria included the following conditions: menstrual bleeding, any prescription drug use and acute or chronic medical illness. The age, sex and smoking habits of the healthy subjects were recorded (Supplemental File 3).

After random urine collection, all of the samples were immediately centrifuged for 30 min at 3,500 g. After precipitate removal, urinary proteins were extracted by acetone precipitation. Lysis buffer (7 M urea, 2 M thiourea, 25 mM dithiothreitol and 50 mM Tris) was used to re-dissolve urinary proteins. The twenty-four urinary protein samples were pooled with equal amounts of protein into one sample for 1D, 2D and 3D analyses (Supplemental File 4).

### GELFrEE and LP-IEF fractionation

For GELFrEE separation, urine samples were prepared using a protocol by Tran *et al*.^45^. Briefly, the pooled sample was fractionated in parallel using an eight-channel multiplexed GELFrEE 8100 Fractionation system (Protein Discovery, Knoxville, TN, USA). Application of 50 V for approximately 75 min and then 100 V for 105 min resulted in twelve GELFrEE fractions. The volume of each fraction was concentrated to approximately 125 μL using a SpeedVac Concentrator (Thermo Fisher Scientific, Asheville, NC, USA). Next, the samples underwent SDS removal using Pierce Detergent Removal Spin Columns (Pierce, Rockford, IL, USA).

For LP-IEF fractionation, urinary proteins were desalted and cleaned using Amicon Ultrafiltration devices with a 10-kDa molecular weight cutoff (Merck Millipore Inc., Billerica, MA, USA). Then, the desalted urinary proteins were focused (approximately 2.5 h at 1 W) using a ten-chamber Microrotofor LP-IEF system (Bio-Rad, Hemel Hempstead, UK). Ten IEF fractions were collected; few protein bands appeared in fractions 7–10. Then fractions 6–10 were pooled into one sample.

### Protein digestion

Urinary proteins were digested with trypsin (Trypsin Gold, mass spec grade, Promega, WI, USA) using filter-aided sample preparation methods^50^. Proteins were loaded onto 10-kDa filter devices (Pall, Port Washington, NY, USA), and 8 M urea in 0.1 M Tris-HCl (pH 8.5) was added to wash the samples. The proteins were denatured by incubation with 50 mM dithiothreitol at 56 °C for 1 h and then alkylated in the dark for 45 min in 55 mM iodoacetamide. Trypsin was added (enzyme to protein ratio of 1:50), and the samples were incubated at 37 °C overnight. After digestion, the peptide mixtures were desalted on Oasis HLB cartridges (Waters, Milford, USA) and lyophilized for high-performance liquid chromatography separation.

### Offline high-pH RPLC separation

In total, nineteen samples, including eighteen fractions that were separated by GELFrEE and LP-IEF and a pooled urine sample, were fractionated by offline high-pH RPLC columns (4.6 mm × 250 mm, C18, 3 μm; Waters Corp, Milford, USA). The samples were loaded onto the column in buffer A1 (10 mM NH_4_FA in H_2_O, pH = 10). The elution gradient was 5–30% buffer B1 (10 mM NH_4_FA in 90% acetonitrile, pH = 10; flow rate = 1 mL/min) for 60 min. The eluted peptides were collected at one fraction per minute. After lyophilization, the 60 fractions were re-suspended in 0.1% formic acid and concatenated into 20 fractions by combining fractions 1, 21, 41 and so on^51^.

### Online LC-MS/MS analysis

Each sample was analyzed on a reverse-phase C18 self-packed capillary LC column (75 μm × 100 mm, 3 μm). The elution gradient was 5–30% buffer B2 (0.1% formic acid, 99.9% acetonitrile; flow rate = 0.3 μL/min) for 100 min. A TripleTOF 5600 coupled with UPLC system was used to analyze the sample, and the MS data were acquired in a high-sensitivity mode using the following parameters: 30 data-dependent MS/MS scans per full scan; full scans were acquired at a resolution of 40,000 and MS/MS scans were acquired at 20,000; rolling collision energy; charge state screening (including precursors with +2 to +4 charge state); dynamic exclusion (exclusion duration 15 s); MS/MS scan range of 250–1800 m/z; and scan time of 50 ms. For 1D separation, the pooled urine sample was analyzed with three technical replicates.

### Data processing

The MS/MS data were processed using Mascot software (version 2.3.02, Matrix Science, London, UK) and searched against the SwissProt database (*Homo sapiens*, 20,267 sequences, 2013_07 version). The search allowed two missed cleavage sites in the trypsin digestion, cysteine carbamidomethylation was set as a fixed modification and both parent and fragment ion mass tolerances were set to 0.05 Da. Mascot search results were filtered using the decoy database method in Scaffold (version 4.3.2, Proteome Software Inc., Portland, OR). Peptide identifications were accepted if they could be shown to achieve a false discovery rate (FDR) of less than 1.0% by the Scaffold Local FDR algorithm. Protein identifications were accepted if they could be shown to achieve a FDR of less than 1.0% and contained at least 2 unique identified peptides. Protein probabilities were assigned by the Protein Prophet algorithm^52^. Proteins that contained similar peptides and could not be differentiated based on MS/MS analysis alone were grouped to satisfy the principles of parsimony. Proteins sharing significant peptide evidence were grouped into clusters.

Total 20 results from 1DLC, 2DLC and 3DLC (12 GELFrEE fractions and 6 LP-IEF fractions) were filtered by Scaffold with the above parameters and yielded average FDRs of 0.10%, 0.26% and 1% at the spectrum, peptide and protein levels, respectively. Then, the 20 datasets were combined together with Scaffold perSPECtives (version 2.0.4, Proteome Software Inc., Portland, OR).

To rank the relative abundance of different proteins, an intensity-based absolute quantification (iBAQ) algorithm was used^53^. The protein intensities were first computed by Progenesis LC–MS (version 2.6, Nonlinear Dynamics, UK)^54^ as the sum of all identified peptide intensities (maximum peak intensities of the peptide elution profile, including all peaks in the isotope cluster). The iBAQ result was obtained as the peptide intensities divided by the number of theoretically observable peptides of the protein (calculated by *in silico* protein digestion; all fully tryptic peptides between 6 and 30 amino acids were counted).

For functional analysis, ingenuity pathway analysis (IPA) software (Ingenuity Systems, www.ingenuity.com) was used to analyze cellular components, canonical gene pathways, functions and candidate biomarkers.

## Electronic supplementary material

Supplemental files

Supplemental Table 1

Supplemental Table 2

Supplemental Table 3

Supplemental Table 4

Supplemental Table 5

Supplemental Table 6
