# Supplementary material for: A comprehensive analysis and annotation of human normal urinary proteome
Source: Sci Rep. 2017 Jun 8;7:3024. doi: 10.1038/s41598-017-03226-6 (PMC5465101; doi:10.1038/s41598-017-03226-6)
Supplement: Supplementary file 1 — Supplemental files [file 41598_2017_3226_MOESM1_ESM.doc]

**A comprehensive analysis and annotation of human normal urinary proteome**

Mindi Zhao^1,2#^, Menglin Li^1,3,#^, Yehong Yang^4^, Zhengguang Guo^4^, Ying Sun^5^, Chen Shao^1^, Mingxi Li^5^, Wei Sun^4,*^ and Youhe Gao^6*^

**Supplemental file legends**

**Supplemental Figure 1.** Tissue distribution and functional annotation of urinary proteins at gene expression level. A) Urinary proteome distributions across 32 tissues. The left numbers in the bracket denote the number of tissue-enriched proteins, the middle numbers denote tissue-enhanced proteins and the right numbers denote group-enriched proteins. B) IPA analysis for the top ten tissues on the basis of tissue-related proteins. Each tissue includes two parts: (1) physiological system function and (2) major pathway. C) The distribution of tissue-related proteins and the corresponding separation strategy for ten tissues.

**Supplemental File 1. Information about identified proteins, peptides, spectra and corresponding FDRs.**

**Supplemental File 2. Twenty-four proteins that cannot be detected by using 3D strategy but can be detected using the 1D and 2D strategies.**

**Supplemental File 3. Individual information for all participants.**

**Supplemental File 4. Protein amounts and total mass spectrometry machine-hour used for 1D, 2D and 3D analyses.**

**Supplemental Table 1. Detailed information about all identified proteins.**

**Supplemental Table 2. All urinary proteins from this study and the proteins from previous urine and exosome proteomic studies.**

**Supplemental Table 3. Relative and estimated concentration of 2,571 proteins from the 2D separation strategy as assessed by iBAQ.** The relative abundance is obtained by multiplying the “iBAQ value” by the molecular weight of the corresponding protein.

**Supplemental Table 4. Estimated concentrations and immunoassay results of 89 proteins.**

**Supplemental Table 5. Detailed information about major pathways of three urine proteome groups.**

**Supplemental Table 6. The information on known biomarkers for predicting various types of organ injury in the Human Urine Proteome Database.**

**Supplemental Figure 1.**


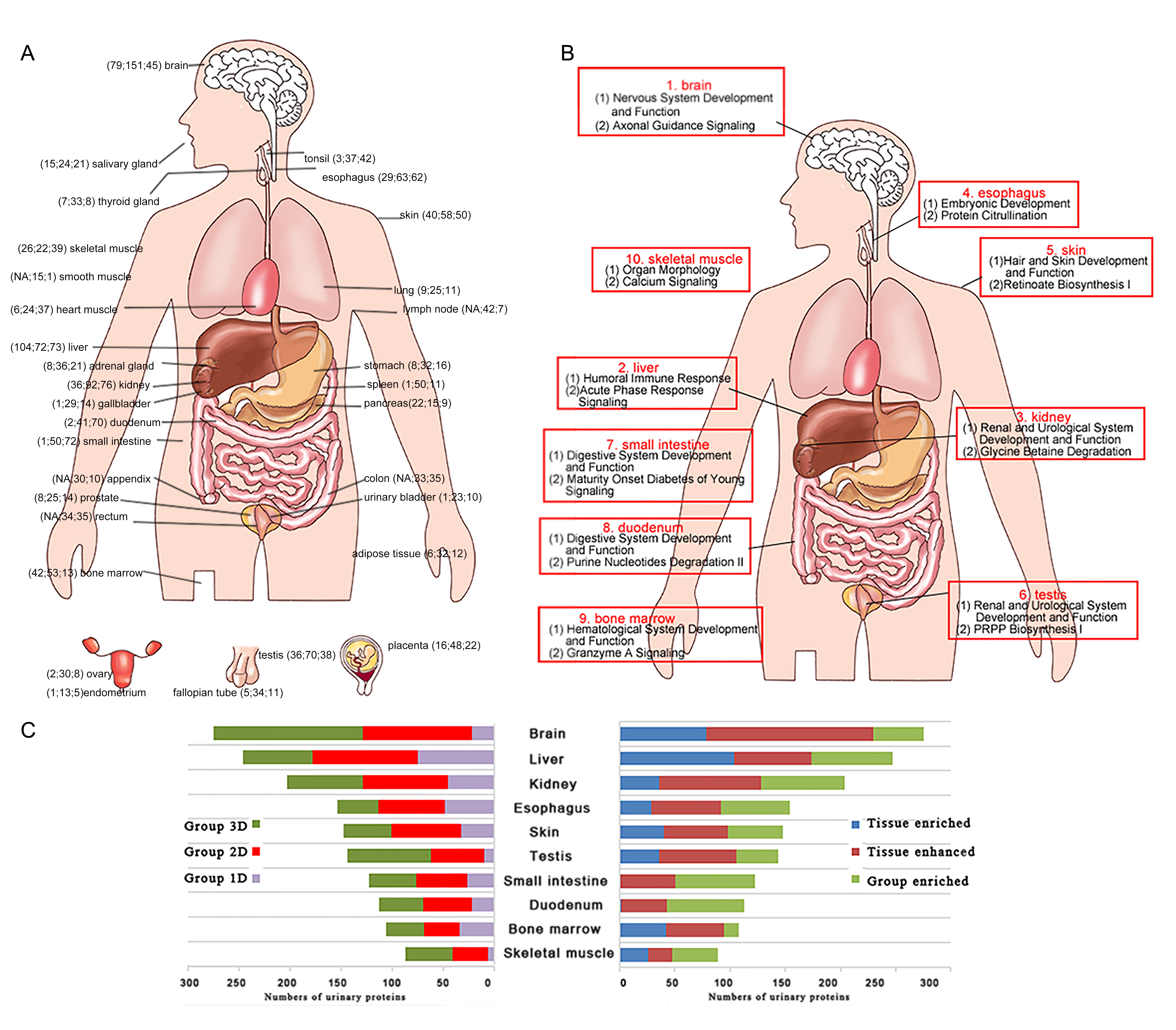


**Supplemental File 1. Information about identified proteins, peptides, spectra and corresponding FDRs.**

|  |  | Numbers | | |  | FDR (%) | | |
| --- | --- | --- | --- | --- | --- | --- | --- | --- |
|  |  | Protein | Peptide | Spectrum |  | Protein | Peptide | Spectrum |
| 1DLC |  | 808 | 13895 | 86517 |  | 0.93 | 0.17 | 0.05 |
| 2DLC |  | 3162 | 25940 | 244854 |  | 0.71 | 0.17 | 0.03 |
| 3DLC | GELFrEE1 | 3103 | 22313 | 200953 |  | 1.06 | 0.31 | 0.07 |
|  | GELFrEE2 | 2568 | 17531 | 134367 |  | 1.12 | 0.35 | 0.14 |
|  | GELFrEE3 | 2574 | 17887 | 121170 |  | 1.03 | 0.31 | 0.11 |
|  | GELFrEE4 | 2875 | 21074 | 171695 |  | 1.11 | 0.33 | 0.11 |
|  | GELFrEE5 | 3048 | 21842 | 148559 |  | 1.08 | 0.31 | 0.15 |
|  | GELFrEE6 | 2669 | 19452 | 147203 |  | 0.96 | 0.28 | 0.07 |
|  | GELFrEE7 | 3319 | 26354 | 179803 |  | 1.05 | 0.29 | 0.06 |
|  | GELFrEE8 | 3226 | 26145 | 209675 |  | 0.72 | 0.19 | 0.07 |
|  | GELFrEE9 | 2548 | 21135 | 158351 |  | 1.10 | 0.28 | 0.09 |
|  | GELFrEE10 | 2535 | 21550 | 178613 |  | 0.97 | 0.24 | 0.07 |
|  | GELFrEE11 | 2718 | 26343 | 177862 |  | 1.01 | 0.23 | 0.05 |
|  | GELFrEE12 | 2034 | 19426 | 119265 |  | 1.00 | 0.22 | 0.07 |
|  | IEF1 | 3201 | 26083 | 166903 |  | 1.03 | 0.26 | 0.29 |
|  | IEF2 | 2288 | 17702 | 144023 |  | 1.12 | 0.29 | 0.06 |
|  | IEF3 | 2249 | 18319 | 135366 |  | 1.04 | 0.26 | 0.06 |
|  | IEF4 | 2241 | 17797 | 114071 |  | 0.95 | 0.26 | 0.06 |
|  | IEF5 | 2281 | 17385 | 108091 |  | 1.03 | 0.27 | 0.16 |
|  | IEF6 | 2239 | 16296 | 101473 |  | 0.99 | 0.28 | 0.13 |
| Final results |  | 6085 | 68151 | 3048648 |  |  |  |  |

**Supplemental File 2. Twenty-four proteins that cannot be detected by using 3D strategy but can be detected using the** 1D and 2D strategies.

| **Uniprot** | **Protein Name** | **1DLC** | **2DLC** | **pI** | **MW** |
| --- | --- | --- | --- | --- | --- |
| P61513 | 60S ribosomal protein L37a | No | Yes | 10.44 | 10144 |
| P62841 | 40S ribosomal protein S15 | No | Yes | 10.39 | 16909 |
| P17096 | High mobility group protein HMG-I/HMG-Y | No | Yes | 10.31 | 11545 |
| Q3SXZ3 | Zinc finger protein 718 | Yes | No | 9.55 | 55405 |
| Q8IU54 | Interleukin-29 | No | Yes | 9.08 | 20018 |
| Q9H3U7 | SPARC-related modular calcium-binding protein 2 | No | Yes | 8.82 | 47436 |
| Q008S8 | Epithelial cell-transforming sequence 2 oncogene-like | Yes | No | 8.65 | 104880 |
| O95857 | Tetraspanin-13 | No | Yes | 8.27 | 22147 |
| P54710 | Sodium/potassium-transporting ATPase subunit gamma | No | Yes | 7.88 | 7283 |
| Q8IX30 | Signal peptide, CUB and EGF-like domain-containing protein 3 | No | Yes | 7.73 | 107238 |
| P62837 | Ubiquitin-conjugating enzyme E2 D2 | No | Yes | 7.69 | 16735 |
| Q6PGP7 | Tetratricopeptide repeat protein 37 | No | Yes | 7.47 | 175355 |
| Q460N5 | Poly [ADP-ribose] polymerase 14 | Yes | No | 6.81 | 202800 |
| Q9H410 | Kinetochore-associated protein DSN1 homolog | Yes | No | 6.57 | 40067 |
| Q6IQ55 | Tau-tubulin kinase 2 | Yes | No | 6.54 | 137412 |
| Q9UHI6 | Probable ATP-dependent RNA helicase DDX20 | No | Yes | 6.49 | 92241 |
| Q8NG11 | Tetraspanin-14 | No | Yes | 6.41 | 30691 |
| Q9Y6X6 | Unconventional myosin-XVI | Yes | No | 6.37 | 206129 |
| A7KAX9 | Rho GTPase-activating protein 32 | Yes | No | 6.27 | 230529 |
| P30685 | HLA class I histocompatibility antigen, B-35 alpha chain | No | Yes | 5.89 | 37804 |
| Q92797 | Symplekin | Yes | No | 5.82 | 141148 |
| Q96M27 | Protein PRRC1 | No | Yes | 5.56 | 46701 |
| Q9Y4F9 | Protein FAM65B | Yes | No | 5.29 | 118519 |
| E2RYF7 | Protein PBMUCL2 | No | Yes | 4.26 | 23830 |

**Supplemental File 3. Individual information for all participants.**

| ID | Gender | Age | BMI | Smoking | Urine column （mL） | Protein concentration （mg/mL） |
| --- | --- | --- | --- | --- | --- | --- |
| 1 | F | 28 | 18.5 | No | 100 | 0.011 |
| 2 | F | 35 | 20 | No | 100 | 0.008 |
| 3 | F | 40 | 21.9 | No | 100 | 0.011 |
| 4 | M | 23 | 19 | No | 100 | 0.022 |
| 5 | M | 45 | 22.3 | No | 100 | 0.029 |
| 6 | F | 32 | 21 | No | 100 | 0.016 |
| 7 | M | 27 | 20.7 | No | 100 | 0.022 |
| 8 | M | 50 | 23.8 | No | 100 | 0.016 |
| 9 | M | 25 | 19.9 | No | 100 | 0.011 |
| 10 | F | 47 | 24 | No | 100 | 0.009 |
| 11 | F | 36 | 21.5 | No | 100 | 0.012 |
| 12 | F | 38 | 22 | No | 100 | 0.022 |
| 13 | F | 50 | 24.6 | No | 100 | 0.011 |
| 14 | M | 33 | 21.3 | No | 100 | 0.017 |
| 15 | M | 57 | 24.3 | No | 100 | 0.005 |
| 16 | F | 37 | 21.3 | No | 100 | 0.010 |
| 17 | M | 43 | 22.6 | No | 100 | 0.015 |
| 18 | F | 21 | 19.8 | No | 100 | 0.014 |
| 19 | M | 57 | 25.2 | No | 100 | 0.013 |
| 20 | M | 39 | 24.1 | No | 100 | 0.018 |
| 21 | F | 29 | 20.5 | No | 100 | 0.020 |
| 22 | F | 39 | 21.2 | No | 100 | 0.024 |
| 23 | M | 60 | 23.1 | No | 100 | 0.025 |
| 24 | M | 35 | 21.3 | No | 100 | 0.026 |

**Supplemental File 4. Protein amounts and total mass spectrometry machine-hour used for 1D, 2D and 3D analyses**.

|  |  | Protein amounts (ug) | MS time (h) |
| --- | --- | --- | --- |
| 1DLC |  | 20 | 2*3 |
| 2DLC |  | 200 | 40 |
| 3DLC | GELFrEE | 5000  3000 | 720  480 |
|  | IEF | 2000 | 240 |
|  |  |  |  |
